# Assessment of the Quality of Life in Patients before and after Coronary Artery Bypass Grafting (CABG): A Prospective Study

**DOI:** 10.3390/ijerph17041417

**Published:** 2020-02-22

**Authors:** Stana Pačarić, Tajana Turk, Ivan Erić, Želimir Orkić, Anamarija Petek Erić, Andrea Milostić-Srb, Nikolina Farčić, Ivana Barać, Ana Nemčić

**Affiliations:** 1 University Hospital Centre Osijek, Osijek 31 000, Croatia; pacaric.stana@kbco.hr (S.P.); turk.tajana@kbco.hr (T.T.); eric.ivan@kbco.hr (I.E.); orkic.zelimir@kbco.hr (Ž.O.); apetek@mefos.hr (A.P.E.); nemcic.ana@kbco.hr (A.N.); 2 Faculty of Medicine, Josip Juraj Strossmayer University of Osijek, Osijek 31 000, Croatia; 3 Nursing Institute “Professor Radivoje Radić”, Faculty of Dental Medicine and Health Osijek, Josip Juraj Strossmayer University of Osijek, Osijek 31 000, Croatia; amsrb@fdmz.hr (A.M.-S.); ibarac@fdmz.hr (I.B.)

**Keywords:** Coronary heart disease, cardiac surgery, coronary artery bypass grafting, risk factors, quality of life, quality-adjusted life-years

## Abstract

The aim of this study was to examine the quality of life and to report on the utility and QALY measures in patients before and after coronary artery bypass grafting (CABG); to investigate whether the SF-12 is comparable with the SF-36 for measuring health-related quality of life of patients with CABG; and to determine the impact of individual predictors on poor quality of life assessment after rehabilitation. This prospective study was conducted between January 2017 and December 2018 at the University Hospital Center Osijek, at three time points: pre-operation, 1 month after surgery, and after rehabilitation. The study was conducted with the SF-36 and SF-12 health questionnaires on 47 participants. After rehabilitation, there was a significant improvement in all domains of quality of life. The highest score was given to the change in pain (BP); mean scores were 63.8 (95% CI 56.9 to 70.6) (*p* = 0.001). The lowest grade (the lowest quality) after rehabilitation was in the domain of limitations due to physical difficulties (RP); arithmetic mean was 48.5 (95% CI 41 to 55.9) (*p* < 0.001). Quality-adjusted life-year was 0.41 (95% CI 0.38–0.44) after the CABG. The results of this study show that patients with coronary heart disease have poor quality of life before surgery. One month after the surgery, the quality of life improved, but was still inadequate. One year after surgery, satisfactory results were obtained in almost all subscales. The SF-36, SF-12, and its components, can be used effectively in patients with CABG. Age, gender, lifestyle, and risk factors in our sample of participants are not predictors of poor quality of life assessment after rehabilitation.

## 1. Introduction

Cardiovascular diseases are one of the leading causes of illness and death in the world. According to the World Health Organization (WHO), around 17.9 million people die annually due to cardiovascular disease worldwide, with an estimated 23 million deaths by year 2030 [1]. According to the data for 2018, in Croatia, 23048 people died from cardiovascular diseases, which makes 43.7% of the total number of deaths, almost half. In the same year, 49% of the women and 38.3% of the men in Croatia died due to cardiovascular diseases [2]. The most common cardiovascular disease is coronary heart disease (CHD), which represents narrowing or clogging of cardiac arteries as a result of atherosclerosis [2]. The most serious complication of CHD is myocardial infarction. 

Risk factors for cardiovascular disease are dyslipidemia, hypertension and diabetes mellitus. Lifestyle habits such as diet, physical inactivity, smoking, age, and gender appear to be fundamental risk factors for cardiovascular disease [3]. These factors have an important influence on cardiovascular risk, which is why their evaluation, treatment and observation are emphasized by the clinical care, research, and treatment guidelines [3]. 

The World Health Organization (WHO) defined health as physical, mental and social well-being. Quality of life implies the ability of people to function normally every day and to be satisfied with their participation in everyday activities. The ability of maintaining these daily activities includes maintaining physical mobility, independence from others, sufficient energy for self-help, social contacts, emotional stability, absence of pain or other symptoms of discomfort, and adequate sleep and rest [4,5]. Coronary artery bypass grafting (CABG) is an important surgical procedure for patients with coronary artery disease, which improves the symptoms, survival and quality of life. Unfortunately, patients’ quality of life after CABG is not improved in all domains, and some patients even experience poorer health-related quality of life (HRQOL) after the surgery [6,7]. Quality of life (QoL) studies differ in the quality of life between pre- and post-surgical treatment. Assessment of post-cardiac surgery QoL involves a comparison of preoperative QoL and postoperative QoL. After CABG, patients often report pain, discomfort, feelings of depression, lack of patience, loss of general well-being, and inability to function at the same level as before the procedure. These feelings can seriously impair the patient’s quality of life [8,9].

In recent decades in the EU, the proportion of the elderly has increased due to low population growth and longer life expectancy. This has increased the incidence of CHD and the number of cardiac surgeries in the elderly. When deciding upon possible surgical treatment in the elderly, the poorer cardiac status of the patient, the greater number of comorbidities, and the greater preoperative risk must be considered [10]. With the improvement and introduction of new operating techniques, lower mortality and morbidity have been reported with respect to the characteristics of older patients [11]. Different models to predict outcomes after CABG have been developed [12]. The quality of life study, measured by the SF-36 or the SF-12, and its components, can be used effectively in patients with CHD [13]. The SF-36 contains 36 items and thus places a considerable burden on patients and investigators [14]. Patients are often exhausted and do not want to fill out a long questionnaire. Therefore, Ware et al. decided to develop a shorter questionnaire, the SF-12 reducing the number of items from 36 to 12, which would take less than 2 min to complete [15].

CABG is not only an extension of life, but also an improvement in functional mobility, quality of life, and maintenance of independent status, indicating that patients have benefited significantly from surgery, as they perceived and reported the QoL results [16]. Improvements in HRQOL, expressed as utility measures and quality-adjusted life-years (QALYs), are also important targets of treatment. Utility scores are used as preference weights to calculate QALYs, which incorporates both the impact of the treatment on a patient’s length of life and the impact on their HRQOL into a single measure [17]. QALYs are recommended as a summary measure of health outcomes [17].

Results from a study conducted in 2004 in Croatia showed that 1 year after surgery, the patient’s health status was significantly improved in half of the physical and mental health domains compared to the pre-surgery status [18]. The aim of this study was to examine the quality of life and to report on the utility and QALY measures in patients undergoing coronary artery bypass grafting (CABG) before, 1 month after surgery, and at one-year follow-up after rehabilitation. The study also tried to investigate whether the short form SF-12, is comparable with its longer version, the SF-36, for measuring health-related quality of life of patients with CABG and to determine the impact of individual predictors on poor quality of life assessment after rehabilitation. 

## 2. Materials and Methods

This prospective study was conducted between January 2017 and December 2018 at three study time points: before surgery, 1 month after surgery, and after rehabilitation (1 year after surgery). The study was conducted at the University Hospital Center Osijek with the approval of the Ethics Committee (R1: 8099-7/2017). All participants were informed and agreed upon the purpose of the research and the anonymity of the data, and their participation was voluntary. The study was conducted with the Croatian version of the SF-36 health questionnaire, the sociodemographic questionnaire (gender, age, education, marital status, smoker status) and data from medical records (diabetes mellitus, hypertension, hyperlipidemia). In addition to the questionnaires, the participants were provided with a written explanation of the survey and written instructions on how to complete the questionnaire. Participants were also asked for written consent to participate in the study.

Patients between 30 to 75 years of age with elective coronary artery bypass surgery, optimal surgical revascularization and ejection fraction greater than 20%, and who were able to speak and read Croatian were included in the study.

Exclusion criteria for the study were emergency patients; patients with palliative revascularization; patients with prolonged intensive care unit stay (longer than 3 days); postoperative neurocognitive dysfunction; ejection fraction less than 20%; patients below 30 and over 75 years of age, with life expectancy of less than 1 year; cognitive and/ or mental illness; illiteracy; and inability to communicate in Croatian.

From the total of 146 CABG procedures, there were 65 patients who met the criteria. During the study, 10 patients withdrew their approval to participate before surgery, while five questionnaires were not correctly completed 1 month after surgery. After rehabilitation, three participants did not respond to a cardiac surgeon follow-up, therefore, the total number of participants in the study was 47. The first measurement was performed on admission to the hospital where patients completed the questionnaire prior to surgery; the second measurement was performed 1 month after surgery at the cardiac surgery follow-up; and the third measurement was performed 1 year after the patients underwent rehabilitation, which lasted 3 weeks.

### 2.1. Sample Size Calculation

To determine the mean effect in the difference of numerical variables between the three measurements performed (preoperatively, postoperatively and after rehabilitation), with a significance level of 0.05 and a power of 0.95, the minimum required sample size is 43 participants (calculation made using G * Power version 3.1. 2, Franz Faul, University of Kiel, Germany).

### 2.2. The SF–36, SF-12 and SF-6D Health Questionnaire

The SF-36 Health Questionnaire [19,20,21] consists of 36 sections (items) that study physical, psychological, and social functioning. Each item refers to one of the eight different health indicators. The SF-36 is a generic physical and mental health measurement questionnaire designed to compare patients with different ailments or patients with different treatments. It is a multidimensional questionnaire composed of 36 items covering eight areas: physical functioning (PF), physical role functioning (RP), physical pain (BP), general health (GH), vitality (VT), social functioning (SF), emotional role functioning (RE), and mental health (MH). The overall result of the first four areas gives a physical health assessment (PCS), and with the other four areas a mental health assessment (MCS). Compared with the SF-36, the SF-12 has only one or two items from each of the eight health concepts of the SF-36 [15]. The SF-12 items allow the calculation of the mental component summary (MCS) and physical component summary (PCS) scales but not of the domains. The scoring algorithms for the summary measures and the items selected for the SF-12 were validated in nine countries [22]. The total score SF-36 is most often presented in the form of a profile defined by eight questionnaire areas that represent the benchmarks of only health assessments transformed into a single scale, whose theoretical minimum score is 0 and maximum 100. In all questionnaire areas, a higher score indicates better subjective health [19,20,21]. Points greater than 50 indicate a preserved or good QOL. The reliability and validity of this instrument have been determined. The Croatian version of the SF-36 questionnaire was used and validated in Croatia [23]. The internal reliability coefficient of the Cronbach Alpha scale before surgery was 0.746, after surgery 0.781, and after rehabilitation 0.813. 

The items of the SF-36 were converted into the QALY using the SF-6Dv2 [24,25]. The SF-6D is a single-index summary preference-based measure of health derived from 10 or 11 items of the SF-36, depending on the version used. The resulting SF-6D index, scored from 0 to 1, where 0 represents worst health state (death) and 1 represents perfect health, can be used in the assessment of the QALYs and the cost-effectiveness of various healthcare interventions. QALYs were estimated by calculating the individual area under the curve of the SF-6D, for the periods between measurements until one year after the CABG.

### 2.3. Statistical Methods

Categorical data are represented by absolute and relative frequencies. The normality of the distribution of numerical variables was tested by the Shapiro–Wilk test. Numerical data are described by arithmetic mean and confidence interval (95% CI). Differences in numerical variables between measurements were tested by ANOVA for repeated measurements (Post-hoc Bonferonni). Logistic regression analysis assessed the impact of multiple factors (gender, age, whether they live alone or not, risk factors) on the probability of lower quality of life after rehabilitation. All P values are two-sided. The significance level was set to Alpha = 0.05. The statistical program MedCalc Statistical Software version 18.2.1 (MedCalc Software bvba, Ostend, Belgium; 2018) and SPSS (IBM Corp. Released 2013. IBM SPSS Statistics for Windows) were used for statistical analysis. Version 21.0. Armonk, NY: IBM Corp.).

## 3. Results

The study was conducted on 47 participants, 26 (55%) male and 21 (45%) female. Most participants were over 55 years of age. In relation to the level of education, 20 (43%) had a high school degree, and 5 (11%) had college or university degree. Thirthy-four (72%) participants were married and 12 (26%) lived alone. According to risk factors, 21 (45%) participants were smokers, 35 (75%) had hypertension, 16 (34%) had diabetes, and 29 (61%) had high cholesterol (Table 1). 

Measured by SF-36, prior to surgery, the best rating was given to social functioning (SF), which did not change significantly over time, although it did rate slightly worse. After rehabilitation, there was a significant improvement in all domains of quality of life, the highest score was given to the change in pain (BP), mean scores were 63.8 (95% CI 56.9 to 70.6) (*p* = 0.001). The lowest grade (the lowest quality) after rehabilitation was in the domain of limitations due to physical difficulties (RP); arithmetic mean was 48.5 (95% CI 41 to 55.9) (*p* <0.001) (Table 2). In the domains of mental health (MH), vitality/ energy (VT) and overall mental health (MH), only the change between pre-surgery and post-rehabilitation measurements was significant, whereas there were no significant differences between pre- and post-surgery measurements (Table 2).

In the physical component summary SF-12 (PCS), there is a significant difference between pre- and post-surgery measurements, before surgery and after rehabilitation. In the mental component summary SF-12 (MCS), significant differences were found between pre-surgery and post-rehabilitation and post-surgery and post-rehabilitation measurements (Table 3).

Table 4 shows differences in the score of scale SF-36 before and after the surgery, as well as after the surgery and after the rehabilitation. There was a strong correlation in differences of the physical and mental component of SF-12, with differences in assessment after operation and after rehabilitation. Greater difference in score SF-36, after surgery and after rehabilitation, correlates with a greater physical component score (PSC) in SF-12 (r = 0.958; *p* < 0.001) and a slightly lower, but also satisfactory mental component score (MSC) in SF-12 (r = 0.925; *p* < 0.001). (Table 4). 

The participants were divided according to the quality of life assessment to those with a poorer quality (score 0–60) and respondents more satisfied with the quality of life (score 61–100). Score of less than 60 had: 45 (95,7%) participants before surgery, 39 (83%) participants 1 month after surgery, and 27 (57,4%) participants after rehabilitation. 

Logistic regression evaluated the impact of individual predictors on poorer quality of life scores after rehabilitation. It was noted that age, gender, lifestyle, and risk factors, in our sample of respondents, were not predictors of poor quality of life assessment after rehabilitation (Table 5).

Statistical differences were not found among the baseline, 1 month after surgery, and 1 year after rehabilitation in SF-6Dv2 utility scores. The mean QALY measurement across the one study year was 0.41 (95% CI 0.38–0.44) for the CABG patients (Table 6). 

## 4. Discussion

This study assesses quality of life pre-operatively, post-operatively and 1 year following rehabilitation on 47 individuals who have had elective CABG surgery in Croatia. The study demonstrates an improvement in most areas of quality of life following either surgery or rehabilitation. Regarding the questionnaires used, the SF-12 is an effective alternative to the SF-36 for the assessment of health-related quality of life of patients with CABG. Quality-adjusted life-year was 0.41 after the CABG. It was observed that age, gender, lifestyle and risk factors, in our sample of participants, are not predictors of poorer quality of life assessment after rehabilitation.

Comparison of our results with 1-year post-CABG findings of four different studies in various parts of the world, conducted in Australia [10], Iran [26], USA [27], UK [28], and one in Croatia 2004 [18] separately show how our assessment of life quality in almost all domains is lower in comparison to other studies. However, in comparison with subscales of Croatian [18] previous study the assessments in this study where higher (PF, RP, BP, GH domains), equal or slightly lower (RE, MH, VT domains). The greatest improvement is in the domain of pain (BP), which can be explained by the fact that currently in Croatia, the pain is treated better and that patients follow pain therapy recommendations. The interesting difference, in the domain of pain (BP), is the study conducted in Iran [26] where the pain was estimated with the lowest score in comparison to this study and all other studies [10,27,28], which was explained with noncompliance with prescribed medications and advices. 

The SF domain in this study is estimated lower and did not change significantly over time in comparison to all other studies [10,26,27,28], and decreased in comparison with the previous Croatian [18] study. That can be explained due to higher rates of comorbidities and the older-aged patients in this study, as well as inadequate support from the community, and that might be a point of direction to act on Croatian patients after CABG. Participants in this study scored a lower mean value 1 year following rehabilitation for all eight health domains assessed by SF-36, compared with the general Croatian population means [23].

These results show how people differ in various parts of the world and can progress in a positive direction, such as in this study compared to the 2004 study in Croatia [18]. This can include different interventions in different countries to improve the quality of life of these patients. Differences between our and other studies can be explained with the fact that the HRQOL would not improve in a linear way for all patients after CABG, and the norms of HRQOL are different in various countries.

Most of our patients were married, so the stabile results of social functioning (SF) in this study are not surprising. Research shows that spousal social support reduces mortality and improves psychosocial recovery after surgery, as patients often rely on their partner for help before and after surgery. The comorbidities of cardiac patients are hyperlipidemia, hypertension, smoking, and diabetes [28,29], and partners give them emotional support and also help them adopt to a healthier lifestyle, reduce risk factors, or stick to their treatment through participation in a comprehensive rehabilitation program [30,31,32]. 

Poor results in our patients before and after surgery in the area of physical functioning (PF), limitations due to physical difficulties (RF), and emotional difficulties (RE) can be explained with limitations in daily activities, which may be related to the inability to work, as well as a number of symptoms of illness and psychological difficulties such as fear, anxiety, postoperative course, and concern for the future, which is in line with other research [33,34].

In this study, after the surgery, patients underwent early rehabilitation and early mobilization by physiotherapists who taught them deep breathing, coughing, and walking exercises to ensure adequate oxygenation, mucus secretion, and prevention of respiratory tract infection, and improved endurance and physical functioning. Early rehabilitation may prevent future complications [35,36]. However, the results of this study showed that patients did not achieve satisfactory quality of life results in the area of physical functioning (PF) after discharge from the hospital, 1 month after surgery.

The results of this research in the area of mental functioning (MH), vitality/ energy (VT), pain (BP), and perceptions of general human health (GH) were low before surgery, whereas post-surgery measurements showed improvement in health assessment and changes in general health. The Health-Related Quality of Life (HRQOL) examination of these patients before and after cardiac surgery showed an improvement 1 year after surgery in the area of physical and mental health compared with pre-surgery status. A study conducted in Brazil on patients who were waiting for CABG surgery estimated that the diagnosis of heart disease and the waiting period for surgery were factors that negatively affected emotional reactions, their behavior, and symptoms of the disease. Most patients state that fear, anxiety, uncertainty about the future, and chest pain limit their daily activities. For these patients, surgery meant the end of deteriorating health and the beginning of a new life [33,37]. The results of a study by Myles and associates [38] showed that not all patients have a better quality of life after surgery; postoperative complications (respiratory difficulties, cardiac arrhythmia, acute renal failure, stroke, wound infection) may impair the quality of recovery after cardiac surgery for up to 3 months. CABG surgery is not only for prolonging the life of patients, it is important for improving the quality of life of heart patients. Therefore, it is important to evaluate pre-operative conditions of cardiac surgery patients to obtain results that can be compared with post-operative quality of life outcomes. 

After the CABG surgery, there was an improvement in general health (GH), which may be related to the reduction or elimination of angina and chest pain [28], which is consistent with the results of this study. After rehabilitation, a significant improvement in all domains of quality of life was observed in our patients, and the highest score was recorded in the pain scale, which is consistent with other research findings [39,40]. Quality of life has been found to deteriorate shortly after the intervention and then moves in a positive direction with patient recovery within a year [41]. However, the results of a study conducted in Poland in patients who had CABG surgery showed that before the start of treatment and rehabilitation, chest pain, dyspnea and fatigue were 68%, 50% and 40% in patients, after rehabilitation these rates reduced to 10%, 12% and 33%. However, patients continued to complain of sleep pain (53%) and leg pain (25%), which could be explained by the short period of time since surgery [42].

After rehabilitation, the lowest score was recorded in limitation due to physical difficulties (RP), but in comparison with the RP subscale of the previous Croatian [18] study, they where higher. This can be explained by the fact that these patients were more than 60 years old, 23 of them (49%), and that there were side effects after surgery and after rehabilitation, and the frequency of risk factors in these patients increased. However, on the other hand, it represents an improvement in the rehabilitation program in Croatia. In Croatia, all patients undertake cardiac rehabilitation programs and usually stay for about 3 weeks in inpatient cardiac rehabilitation for a 12-month period after CABG. The Croatian healthcare system covers all costs of rehabilitation, and rehabilitation is available to all. The cardiac rehabilitation program is a comprehensive program that includes physical activity, exercise, nutrition and psychological counseling, blood pressure control, cholesterol and blood glucose control, and smoking cessation. This is a type of program that recommends the rehabilitation and improvement of the quality of life in patients after open heart surgery, as well as for the prevention of future complications [35,36]. In a study conducted by Unsar and associates [43] comparing HRQOL in patients with and without coronary diseases, the results showed that patients with coronary diseases have lower results in physical difficulties such as mobility, elimination, hearing, breathing, physical activity, and sexual vitality functionality, which can affect their postoperative functional status. As a result of a change in health, it has been reported following rehabilitation that it can relieve pain, reduce depression, and help patients perform daily physical activities to relieve symptoms of other illnesses associated with CHD and surgery, which are all in line with the results of other studies [34,44,45]. 

In this study, the differences in the change in the rating of individual domains of quality of life compared to the time of measurement were evaluated. Significant results were observed for pre- and post-rehabilitation measurements, whereas pre- and post-operative measurements showed no significant differences. In these patients, an HRQOL assessment measured at a single point in time before surgery can differentiate the level of health in patients during a recurrent time after one year. 

Various questionnaires are used to examine the quality of life of patients with CHD. Dempster and Donnelly [46] compared the SF-36 with other generic questionnaires such as the Nottingham health profile and others for patients with coronary disease. They concluded that the SF-36 is the most appropriate instrument to assess HRQOL of cardiac patient populations. Howver, it contains 36 items and thus places a considerable burden on patients. The SF-12 reduced the number of items to 12, which takes less than 2 min to complete [15]. The SF-12 summary scores were in high correlation with the SF-36 summary scores for patients with CABG in this study, which is consistent with results of the study carried out in Germany on patients with CHD [13]. The SF-36 and SF-12 and its components can be used effectively in patients with CABG.

The results of this study show that the comorbidities and risk factors of these patients are hyperlipidemia, hypertension, smoking, and diabetes, and they are similar to the results of other studies [28,29]. It was noted that age, gender, lifestyle and risk factors, in our sample of participants, are not predictors of poorer quality of life assessment after rehabilitation, which is contrary to the results of other studies [26,28,29]. This may be explained by the fact that the participants in this study rated their quality of life worse than the participants in similar studies in other countries [10,26,27,28,29]. The items of the SF-36 were converted into the QALYs (using the SF-6Dv2) [24,25], and quality-adjusted life-year was 0.41 after the CABG in this study. Comparison of our results with 1-year post CABG findings of other different studies shows how our results are lower in comparison to other studies 0.69 [47], 0.77 [48] and 0.79 [49], which is not surprising considering the results of quality of life in our study. These results indicate a poor quality of life assessment, regardless of risk factors, and it is likely that all cardiac patients have almost all or most of the risk factors, which was the case in this study; regarding hypertension medication, 75% are examinees, 61% have high cholesterol, and 45% are smokers.

The limitation of this research is the small sample size and the conduction at a single center in one geographical area. One of the limitations of this study is that we do not have adequate data for economic evaluation, which will certainly be the subject of our further research. Patients undergoing emergency surgery were excluded from the study because they may not be able to provide accurate information about their perception of their health status prior to surgery, which may also be a limitation of this research. Patients undergoing palliative revascularization were excluded from the study, which might also be a limitation to this study since we could not obtain quality of life data in patients with optimal revascularization compared to patients with palliative revascularization. The results of our study cannot be generalized to the entire population of patients who have had CABG surgery in Croatia, but they can serve to design interventions that will improve the quality of life of these patients.

## 5. Conclusions

The results of this study show that patients with CHD before surgery have a poor quality of life, with low scores in all subscales except for social functioning (SF). After surgery, good quality of life is manifested in the subscales of perceptions of general health (GH) and changes in health, while other subscales, although better than before surgery, still indicate an unsatisfactory quality of life. One year after surgery, that is, after rehabilitation in these patients, satisfactory results were obtained in all subscales except in limitation due to physical difficulties (RP), vitality/ energy (VT) and social functioning (SF).

The results of this study showed an improvement in general health status after surgery and after rehabilitation in these patients, which is consistent with other studies [10,18,26,27,28,34,39,50]. However, although there has been an improvement in quality of life 1 year since surgery, the results indicate an unsatisfactory quality of life for patients after CABG surgery. Quality-adjusted life-year was 0.41 after the CABG. The SF-36 or the SF-12 and its components can be used effectively in patients with CABG. Age, gender, lifestyle, and risk factors, in our sample of participants, are not predictors of poor quality of life assessment after rehabilitation. 

## Figures and Tables

**Table 1:** Basic characteristics of the participants.

	*n* (%)
Gender	
Male	26 (55)
Female	21 (45)
Age groups	
30–45 years	2 (4)
46–50 years	4 (9)
51–55 years	3 (6)
56–60 years	15 (32)
Older than 60 years	23 (49)
Education levels	
Unfinished elementary school	4 (8)
Elementary school	13 (27)
High school	20 (43)
College	5 (11)
University	5 (11)
Marital status	
Single	1 (2)
Married	34 (72)
Divorced	5 (11)
Widow/widower	7 (15)
Lives alone	12 (26)
Smokers	21 (45)
On hypertension medication	35 (75)
Diabetics	16 (34)
Have high cholesterol	
Yes	29 (61)
No	13 (28)
Unknown	5 (11)

**Table 2:** Self-assessment of the SF-36 quality of life domain and components before and after surgery and after rehabilitation.

SF-36	Mean Value (95% CI)			p*
	Before Surgery	After Surgery	After Rehabilitation	
†Physical functioning (PF)	23 (15.2–30.8)	49.7 (42.1–57.3)	56.7 (49.2–64.3)	<0.001
†Role–physical (RP)	28.9 (22.5–35.3)	44.6 (37.3–51.9)	48.5 (41.0–55.9)	<0.001
†Role–emotional (RE)	32.1 (24.9–39.1)	46.6 (40.1–53.1)	54.9 (47.4–62.4)	<0.001
Social functioning (SF)	51.9 (47.2–56.6)	50.6 (46.4–54.8)	49.7 (44.5–54.9)	0.51
‡Mental health (MH)	47.3 (42.1–52.4)	49.8 (45.3–54.2)	57.3 (51.8–62.7)	0.009
‡Vitality/ energy (VT)	39.4 (34.1–44.7)	43.5 (39.2–47.8)	49.5 (44.9–54.0)	0.003
§Bodily pain (BP)	45.8 (39.2–52.3)	49.5 (43.3–55.7)	63.8 (56.9–70.6)	0.001
†General health (GH)	41.7 (34.6–48.8)	52.1 (46.9–57.2)	53.9 (48.9–58.8)	0.003
‡Health changes	42.1(32.0–52.1)	54.3(44.6 –63.9)	59.1(52.4–65.9)	0.004
† Physical component summary (PCS)	35.6 (30.9–40.5)	48.7 (43.9–53.4)	56.1 (51.1–61.0)	<0.001
‡ Mental component summary (MCS)	42.6 (39.1–46.1)	47.0 (43.8–50.3)	52.9 (49.3–56.6)	0.02

Legend: *Repeated measures of analysis of variance, Post hoc Bonferroni; †on level of *p* < 0.05 significant differences between before and after surgery, before surgery vs. after rehabilitation; ‡on level of *p* < 0.05 significant differences between before surgery vs. after rehabilitation; §on level of *p* < 0.05 significant differences between before surgery vs. after rehabilitation, after surgery vs. after rehabilitation.

**Table 3:** Self-assessment of the SF-12 quality of life components before and after surgery and after rehabilitation.

SF-12	Mean Value (95% CI)			p*
	Before Surgery	After Surgery	After Rehabilitation	
†Physical component summary (PCS)	32.5 (27.6–37.4)	42.7 (37.4–48.7)	51.9 (46.3–57.5)	<0.001
§Mental component summary (MCS)	38.8 (33.7–43.9)	40.9 (35.9–45.9)	50.9 (45.4 – 56.5)	0.002

Legend: *Repeated measures of analysis of variance, Post hoc Bonferroni; †on level of *p* < 0.05 significant differences between before and after surgery, before surgery vs. after rehabilitation; §on level of *p* < 0.05 significant differences between before surgery vs. after rehabilitation, after surgery vs. after rehabilitation.

**Table 4:** The connection of component differences (post-surgery—pre-surgery; post-rehabilitation— post-surgery) SF-36 and SF-12.

	Pearson’s Correlation Coefficient r (*p* Value) SF-36	
	Difference between Post-Surgery–Pre-Surgery	Difference between Post-Rehabilitation–Post-Surgery
SF-12		
Physical component summary	0.863 (<0.001)	0.958 (<0.001)
Mental component summary	0.901 (<0.001)	0.925 (<0.001)

**Table 5:** Predicting the probability of poorer quality of life after rehabilitation (univariate regression analysis).

Predictor	β	St. Error	Wald	P	OR	95% CI
Physical component summary						
Age groups (up to 55 years)						
56–60 years	0.69	0.95	0.53	0.47	2.0	0.31–12.8
Older than 60 years	1.61	0.89	3.24	0.07	5.0	0.87–28.7
Lives alone	2.13	1.12	3.6	0.06	8.4	0.94–75.1
Smokers	0.20	0.66	0.09	0.76	1.2	0.33–4.49
Mental component summary						
Age groups (up to 55 years)						
56–60 years	0.59	0.98	0.35	0.55	1.8	0.26–12.5
Older than 60 years	0.88	0.92	0.91	0.34	2.4	0.39–14.6
Gender (female)	0.41	0.74	0.29	0.59	1.5	0.35–6.42
Diabetics	0.69	0.74	0.87	0.35	2.0	0.47–8.56

**Table 6:** SF-6Dv2 utility score.

Period	SF-6Dv2 Utility Score		*p* *
	Mean	95% Cl	
Baseline/before surgery	0.442	0.411 to 0.473	0.10
One month after surgery	0.436	0.406 to 0.465	
One year after surgery/after rehabilitation	0.414	0.380 to 0.448	

* Repeated measures of analysis of variance.
